# Epigenetic mechanisms of plasticity and resistance in glioblastoma: therapeutic targets and implications

**DOI:** 10.3389/freae.2025.1519449

**Published:** 2025-05-16

**Authors:** Farzaneh Amirmahani, Saurav Kumar, Sree Deepthi Muthukrishnan

**Affiliations:** Department of Oncology Science, University of Oklahoma Health Sciences Center, Oklahoma City, OK, United States

**Keywords:** GBM, epigenetic, DNA methylation, histone modifications, non-coding RNAs, GBM stem-like cells, phenotypic plasticity, therapeutic resistance

## Abstract

Glioblastoma (GBM), a highly aggressive and malignant form of primary adult brain cancer, poses significant therapeutic challenges. Despite our improved understanding of the cellular and molecular mechanisms underlying tumorigenesis and the evolution of GBM, targeted molecular therapies have failed to improve patient survival outcomes. The failure of standard treatments and targeted therapies is mainly attributed to the acquisition of phenotypic plasticity of tumor cells and GBM stem-like cells. Epigenetic modifications and their mediators have emerged as crucial regulators of phenotypic plasticity, influencing tumor heterogeneity, therapy resistance and disease progression. Here, we summarize and provide insights into epigenetic regulation of GBM plasticity and specifically, focus on the roles played by DNA- and histone modifiers and non-coding RNAs in driving phenotypic plasticity and resistance. We also delve into their dynamics in response to standard therapies and the challenges for targeting them to overcome phenotypic plasticity and resistance in GBM.

## Introduction

Glioblastoma (GBM) is the deadliest and most common primary adult brain tumor with a median survival of 14–15 months post-diagnosis. Tumors are highly refractory to standard treatments of surgical resection, fractionated radiation and chemotherapy with temozolomide (TMZ) (Wu et al., 2021). This results in near universal recurrence of GBM in all patients, indicating the critical need for better therapeutic strategies. The aggressive behavior and treatment resistance of GBM is primarily attributed to the presence of a small subset of tumor cells with stem-like properties, the GBM stem-like cells (GSCs). GSCs can self-renew and differentiate into multiple lineages by reactivating developmental transcriptional programs and signaling pathways. GSCs exhibit metabolic adaptability, increased tumorigenic properties, enhanced DNA repair capacity and chemo-radiation resistance compared to non-stem cancer cells (Prager et al., 2020). Furthermore, GSCs also exhibit cellular and lineage flexibility, transdifferentiating into different cell types, particularly, endothelial-like cells (EC) and pericyte-like cells (PC), the two major cellular components of the blood vessels (Ricci-Vitiani et al., 2010; Cheng et al., 2013). These GSC-derived EC and PC integrate into the vasculature, aid in the maintenance of blood-tumor-barrier and tumor growth (Cheng et al., 2013; Zhou et al., 2017).

Although standard chemo-radiation therapy can eliminate proliferating tumor cells, the therapeutic stress can reprogram the non-stem tumor cells to acquire a multipotent status. This multipotent status allows tumor cells to dedifferentiate and acquire stem-like characteristics, become GSC-like giving rise to therapy-resistant clonal populations that contribute to tumor recurrence (Bhat et al., 2020). Radiation therapy has been shown to promote the transition of GSCs from a proneural-(PN) to a mesenchymal (MES)-like state (PMT) similar to epithelial-mesenchymal transition (EMT) in other cancers (Minata et al., 2019; Shibue and Weinberg, 2017). These radiation-induced MES-like GSCs display increased capacity for invasion, therapeutic resistance, and poor prognosis (Minata et al., 2019). Furthermore, a recent study demonstrated that radiation therapy induces the transdifferentiation of GSCs into both EC-like and PC-like cells and these transdifferentiated cells provide a trophic niche to support tumor recurrence (Muthukrishnan et al., 2022). TMZ chemotherapy has also been reported to promote GSC transdifferentiation in ECs (Baisiwala et al., 2019). These studies support the prevailing notion that sandard chemo-radiation therapy acts as a double-edged sword in that they eliminate proliferating tumor cells but induce plasticity in surviving tumor cells and GSCs leading to therapeutic resistance and recurrence.

The mechanisms contributing to GSC plasticity include cell-intrinsic and extrinsic factors, their localization to hypoxic, invasive or perivascular niches and interactions with the stromal cells in the tumor microenvironment (TME) (Uribe et al., 2022). For instance, the hypoxic niche, characterized by low oxygen levels and overexpression of hypoxia-inducible factors (HIFs), promotes GSC maintenance, chemoresistance, and proneural to mesenchymal-like transition (PMT) (Bar et al., 2010; Joseph et al., 2015). Specifically, HIF-1α promotes glucose uptake and the conversion of pyruvate to lactate, which contributes to the acidic pH (Lu et al., 2002). This altered environment not only supports the metabolic needs of the tumor but also induces PMT in GBM cells, allowing them to adapt and resist therapy (Joseph et al., 2015; Shen et al., 2015). While the hypoxic niche is abundant in MES-like GSC, the invasive niche is enriched with PN-GSCs. Radiation treatment induces the phenotypic transition of the invasive PN-GSCs to MES-like state (Minata et al., 2019). The perivascular niche also regulates GSC response to chemo-radiation therapy via activation of signaling pathways such as NOTCH1 (Guichet et al., 2014). Moreover, the tumor-associated macrophages/microglia (TAM) and reactive astrocytes also promote stemness and PMT of GSC and tumor cells by secreting proinflammatory cytokines like CCL20 and IL-6/8, and activating NF-κB and YAP/TAZ signaling, which in turn induce the expression of mesenchymal proteins and contribute to tumor recurrence (Henrik Heiland et al., 2019; Chen and Hambardzumyan, 2021).

A growing body of evidence has implicated a key role for epigenetic alterations and reorganization of the chromatin structure as a major driver of phenotypic plasticity in GBM. Particularly, hypoxia and radiation are reported to alter DNA methylation and histone modifications allowing GSCs to dynamically shift between phenotypic states (Uribe et al., 2022). Since epigenetic modifications drive the phenotypic adaptability and lineage plasticity of GSC and these alterations are reversible, in principle, the proteins and enzymes of the epigenetic machinery are deemed as promising therapeutic targets to prevent GBM recurrence. This review will focus primarily on the epigenetic regulators associated with phenotypic plasticity and therapeutic resistance in GBM. First, we will provide an overview of the recent findings from single-cell studies examining GSC plasticity, followed by a comprehensive discussion of the epigenetic mechanisms of DNA methylation, histone modifications and non-coding RNAs (ncRNAs) involved in regulating phenotypic plasticity and treatment resistance in GBM.

## Single-cell studies of cellular states and plasticity in GBM

Advances in single-cell sequencing technologies over the last decade have enabled a deeper underst anding of the inter- and intra-tumor heterogeneity of GBM, the complexity and diversity of cell populations that contribute to tumor evolution and therapeutic resistance. Patel et al., conducted the first single-cell RNA sequencing study in primary GBM tumors to investigate intra-tumoral heterogeneity and revealed the existence of multiple molecular subtypes and cellular states within an individual tumor. They noted that these cellular states are associated with diverse transcriptional programs related to oncogenic signaling, stemness, immune response and hypoxia (Patel et al., 2014). Subsequently, the study by Darmanis et al., examined intra- and inter-tumoral heterogeneity by isolating tumor cells from the core and peripheral regions of primary GBM tumors. This study reported significant genomic and transcriptomic diversity within the tumor core, whereas they found a more uniform gene signature in infiltrating tumor cells across patient tumors (Darmanis et al., 2017). A landmark study by Neftel et al., using a large cohort of patient tumors showed that tumor cells exist in four major cellular states: neural progenitor (NPC), oligodendrocyte progenitor (OPC) cell-, astrocytic (AC)- and mesenchymal (MES)- like states and that these cellular states are influenced by specific genomic alterations (Neftel et al., 2019). Other studies utilizing patient-derived orthotopic xenograft (PDOX) and *in vitro* models also demonstrated that GSCs exhibit plasticity and their heterogeneity arises from reversible state transitions and is influenced by the hypoxic environment (Dirkse et al., 2019). Another study utilizing a combination of single-cell RNA- and ATAC-sequencing of primary GBM tumors demonstrated that GSC heterogeneity exists along a single axis of variation and follows a continuum of PN to MES states (Wang et al., 2019). Furthermore, recent studies comparing GBM tumor cells with normal human fetal and adult brain cells revealed that GBM tumorigenesis recapitulates human brain development. These studies showed that the majority of cycling cells are derived from glial-progenitor-like cells and there is an invasive population of GSC with an outer radial glia-like phenotype observed during normal human brain development (Bhaduri et al., 2020; Couturier et al., 2020). Figure 1 provides an overview of the cellular states observed in GBM tumors in response to therapy and by the tumor microenvironment.

**FIGURE 1:**
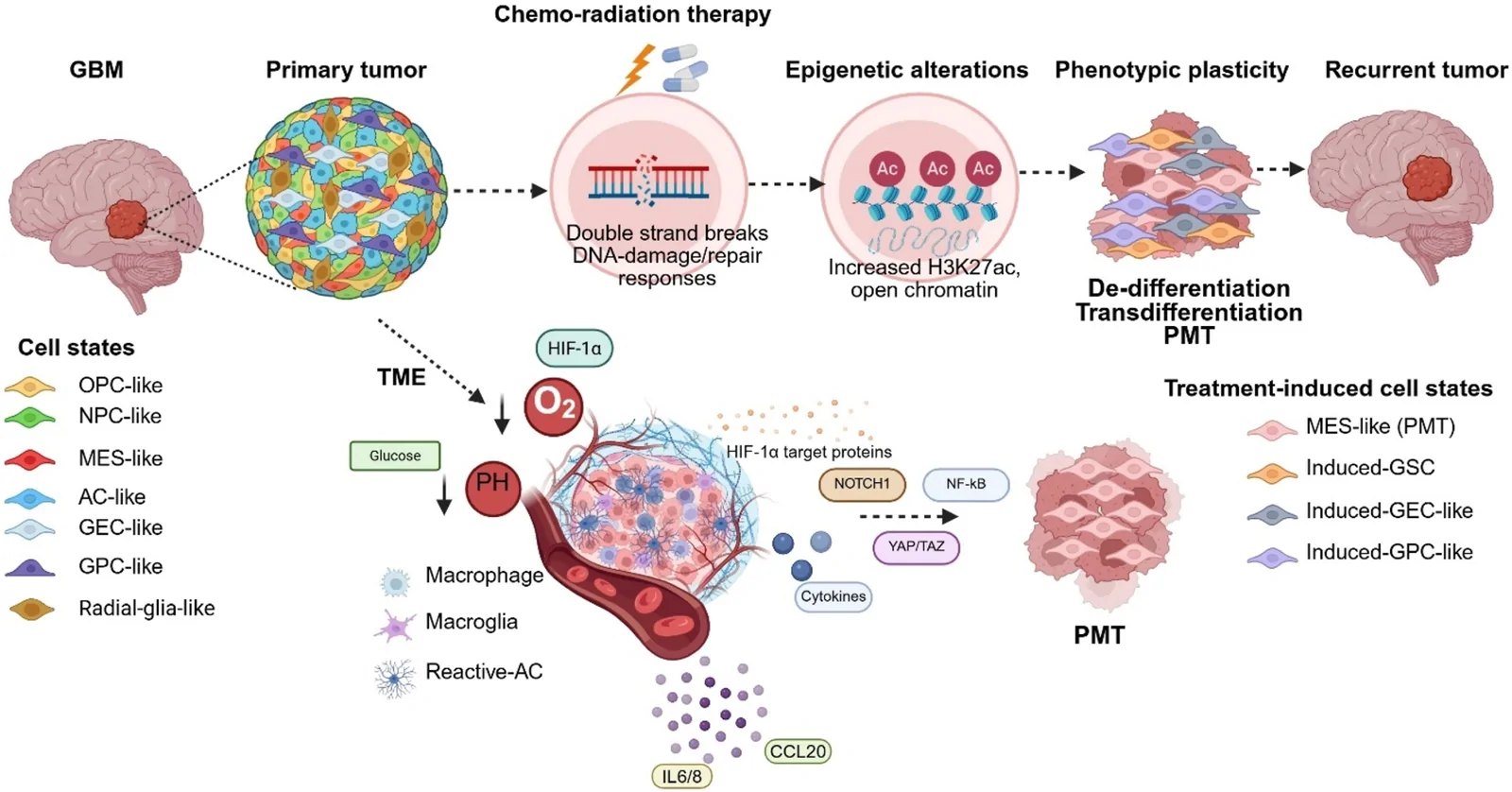
Cellular states and phenotypic plasticity of GSC. Proneural to Mesenchymal transition (PMT) is mediated by chemical cues such as hypoxia, acidic pH, chemo-radiation therapy and interactions with tumor-associated macrophages/microglia, reactive astrocytes. Radiation therapy also promotes phenotypic plasticity by promoting PMT, de-differentiation of tumor cells to GSC-like states and transdifferentiation of GSC into vascular-like cells. (OPC-Oligodendrocyte-progenitor cell, NPC- Neural Progenitor Cell, MES-Mesenchymal, AC-Astrocyte, GEC- GBM-derived Endothelial Cell, GPC-GBM-derived Pericytes).

There are also an increasing number of studies exploring the epigenetic landscape of GBM given the strong evidence of a developmental regulation of GSC. Guilhamon et al., performed single-cell ATAC-sequencing of primary GBM tumors to map the chromatin accessibility of GSC and identified that GSC exist in three states: reactive, constructive and invasive. These cell states possess unique transcriptional signatures and were found in varying proportions within tumors (Guilhamon et al., 2021). Another study by Lu et al., compared the epigenetic landscape of murine and human GSC cultures and found that they aligned along the PN-MES axis and proposed that epigenetic control of GSC is dictated by their developmental origin (Lu et al., 2022). More recent studies have begun to explore the spatial heterogeneity of GBM identifying novel cell types. Jain et al., combined single-cell RNA sequencing and spatial transcriptomics to elegantly demonstrate for the first time the presence of cancer-associated fibroblasts (CAFs) within GBM, which were thought to be absent due to the lack of brain fibroblasts. They demonstrated that CAFs have pro-tumoral effects on GSC but not non-stem tumor cells and promote tumor growth (Jain et al., 2023). In summary, these single-cell transcriptomic and epigenomic studies have illustrated the complexity of cellular states and plasticity of GSC that contributes to treatment resistance and recurrence in GBM.

## Epigenetic mechanisms of GBM plasticity and resistance

The ability of GSC and tumor cells to adapt to diverse microenvironments and persist in the face of treatment requires them to undergo reversible transitions between various cellular states. This transition between different cellular states termed “phenotypic plasticity” is driven by the dynamic restructuring of the transcriptional programs unique to each cell state. Epigenetic modifications play a crucial role in this process as they are reversible events that do not modify the DNA and allow the GSCs to rapidly and efficiently turn on or turn off genes. DNA- and histone methylation is one of the most well-studied epigenetic mechanisms in GBM (J Dabrowski and Wojtas, 2019). However, recent studies have indicated that other histone modifications as well as non-coding RNAs (ncRNAs) play key roles in regulating GBM plasticity and resistance (Azab, 2023; Yin et al., 2013). For instance, radiation-induced transdifferentiation of GSCs is driven by increased histone acetylation in vascular gene regions. Blocking the histone acetyltransferase (HAT) activity of P300 reversed these epigenetic changes, prevented transdifferentiation, sensitized tumors to radiation and reduced tumor growth (Muthukrishnan et al., 2022). Another example is the repressive methylation of miRNA-148a by DNA methyltransferases, which contributes to GSC maintenance and PMT (Li et al., 2019a). In addition, the H3K27 trimethylation (H3K27me3) on promoters of Nanog and PAX6 promotes GSC enrichment and endothelial differentiation, whereas active H3K27ac on the promoters of WNT5A and DLX5 enhances GSC differentiation between proliferative and slow-cycling states (Liau et al., 2017).

## DNA methylation

In GBM, DNA methylation is strongly associated with predicting response to chemotherapy efficacy (Hegi et al., 2005). Early studies determined that methylation of the O(6)-methylguanine-DNA methyltransferase (MGMT) gene promoter as a valuable prognostic biomarker for responsiveness to TMZ therapy (Zhao et al., 2018).

Methylation involves the transfer of a methyl group from S-adenosyl-L-methionine to the C5 position of cytosine residues in the DNA, resulting in the formation of 5-methylcytosine (5mC) (Okano et al., 1998). This process is catalyzed by enzymes called DNA methyltransferases (DNMTs) and include DNMT1, DNMT2 and DNMT3 (DNMT3a and DNMT3b). DNMT1 is involved in methylation maintenance and extension including non-CpG sites, while DNMT2 targets tRNA and DNMT3a and DNMT3b function as *de novo* methyltransferases that methylate CpG (Zhang and Xu, 2017). Several DNMTs including DNMT1 and DNMT3b are highly expressed in GBM and their dysregulation is associated with aberrant cell cycle progression and maintenance of genomic stability (Rajendran et al., 2011). Cheray et al., demonstrated that disruption of specific interactions of DNMT1 with histone modifying enzymes can either suppress or enhance tumorigenesis in a murine GBM model (Cheray et al., 2013). In another study, they showed that inhibition of the DNMT1 interaction with CpG binding protein 1 (CFP1), a member of histone methyltransferase complex, increased sensitivity to TMZ chemotherapy (Cheray et al., 2014). Moreover, DNMT1 and DNMT3b have been shown to be activated by reprogramming transcription factors, OCT4 and SOX2, which leads to global changes in DNA methylation and downregulation of miRNAs that inhibits GBM stemness and tumor-propagating potential (Lopez-Bertoni et al., 2015).

In addition to DNMTs, demethylases such as ten-eleven translocation (TET) dioxygenases (TET1, TET2 and TET3) catalyze the oxidation of 5-methyl deoxycytosine (5-mdC) to 5-hydroxymethyl-2′deoxycytidine (5-hmdC), 5-formly-2′deoxycytidine (5-fdC) and 5-carboxyl-2′deoxycytidine (5-cadC) at promoters and enhancers in a replication-independent manner leading to transcriptional reactivation (Ito et al., 2010; Ito et al., 2011). TET2 overexpression has been shown to regulate neural differentiation and inhibit GBM tumor growth (García et al., 2018), while epigenetic repression of TET3 promotes GBM tumorigenesis (Carella et al., 2020). Furthermore, repression of TET2 by SOX2 and loss of 5hmdC and 5mdC levels is associated increased malignancy and GSC stemness (Lopez-Bertoni et al., 2022). Recent bulk- and single-cell multiomic studies examining global DNA methylation patterns in longitudinal patient samples identified that methylation patterns can be predictive of immune cell infiltration, the extent of necrosis and also patient survival (Klughammer et al., 2018). In addition, DNA methylation was reported to be elevated in aggressive tumors and tightly linked with transcriptional disruption and altered by hypoxia and radiation-stress responses (Johnson et al., 2021). Targeting DNA methylation and DNMTs with small molecule inhibitors such as 5-aza-2′-deoxycytidine, Decitabine and Phthalimido-alkanamide have shown potent anti-tumoral effects and sensitized GBM cells to chemo-radiation therapy in animal models, making it an attractive strategy to overcome therapeutic resistance (Kratzsch et al., 2018; Yamashita et al., 2019; Wee et al., 2019; Gallitto et al., 2020).

## Histone modifiers

Post-translational modifications (PTMs) of histones can influence chromatin structure, gene expression and the transcriptional landscape of cells (Kumari et al., 2023). Several histone PTMs have been identified and include acetylation, methylation, phosphorylation, ubiquitination, lactylation, sumoylation, neddylation, citrullination, ADP-ribosylation, crotonylation, etc. Of these modifications, histone methylation and acetylation are well-characterized in GBM (McCornack et al., 2023). Other histone modifications like ubiquitination, sumoylation, lactylation and phosphorylation are less well-studied. However, these modifications are frequently found in GBM cells and have been linked to poor survival, tumorigenesis and resistance to TMZ (Cheng et al., 2020; Zhou et al., 2019; Wu et al., 2014; Pacaud et al., 2015; Tao et al., 2013).

## Histone acetyltransferases and histone deactylases

Acetylation of histones is mediated by the action of lysine/histone acetyltransferases (KATs/HATs) that catalyze the addition of acetyl groups to N-terminal domain lysine residues, whereas the removal of acetyl groups is catalyzed by histone deactyleases (HDACs). HATs are divided into subgroups based on the their structure and sequence homology and include the Gcn5-related N-acetyltransferase (GNAT)-, MYST- and p300/CBP families as well as nuclear receptor coactivators (SRC-1, ACTR, TIF2), TAFII250 and TFIIIC (Sterner and Berger, 2000). Compared to other histone modifying enzymes, HATs are less well-studied in GBM. Nevertheless, they have been shown to play important roles in regulating GBM plasticity and resistance. Bhat K et al., revealed that radiation induced de-differentiation of tumor cells to GSC-like state is driven by alterations in histone acetylation and methylation, and extensive chromatin remodeling in promoters of Yamanaka factors SOX2, OCT4 and NANOG (Bhat et al., 2020). Muthukrishnan et al., also showed that radiation treatment increases H3K27 acetylation and chromatin accessiblity in specific vascular gene regions to promote GSC transdifferentiation to vascular-like cell states (Muthukrishnan et al., 2022). In another study, Mladek et al., identified that the RBBP4/P300 complex regulates transcription of DNA repair genes via histone acteylation and sensitizes GBM cells to TMZ chemotherapy (Mladek et al., 2022).

While HATs are less well-studied but play a crucial role in promoting plasticity, HDACs have been extensively investigated in GBM resistance. They are grouped into four main classes. Class I HDACs (HDAC1, 2, 3 and 8) have been implicated in GBM, while classes II (HDAC4, 5, 6, 7, 9 and 10) and IV (HDAC11) are over-expressed in low-grade astrocytoma (Lucio-Eterovic et al., 2008). Class III HDACs comprise the sirtuin (SIRT) family of proteins and have been shown to be aberrantly expressed in different GBM cell lines (Kunadis and Piperi, 2022). HDACs 1, 2, 3, 6 and 8 are all associated with TMZ resistance (Yang et al., 2020; Hanisch et al., 2022). SIRT1 was also shown to enhance TMZ resistance in both human GBM lines and xenograft models (Li et al., 2019b). While the investigation of HAT inhibitors is challenging due to their dual roles as both oncogenes and tumor suppressors and lack of substrate selectivity, several HDAC inhibitors (HDACis) have been widely investigated in GBM. HDACis sensitize GBM to radio-chemotherapy and have also been tested in combination with immunotherapies. However, clinical trials using various HDACis have yielded mixed results, with some showing only modest benefits and others displaying disappointing results due to unanticipated toxicity (Everix et al., 2023). Table 1 outlines HAT and HDAC inhibitors investigated in pre-clinical models of GBM as well as in clinical trials.

**TABLE 1:** HDAC and HAT inhibitors investigated in GBM cell lines and in clinical trials.

Drugs	Targets	Cell lines/Clinical trials	Outcomes	References
Vorinostat (SAHA)	Class I and II HDACs	U87-MG cells and GSC lines	Radiosensitization	Pont et al. (2015), Berghauser Pont et al. (2014), Menezes et al. (2019)
Tinostamustine	Class I, II and IV HDACs	U251MG, U373, U118, U138, A172, U87MG, LN19, SW1783, SNB19, LN229, T98G and D54	Synergistic effects with radiotherapy	Festuccia et al. (2018)
Trichostatin A	Class I and II HDACs	U87-MG, U373, U251 and Hs683	Radiosensitization, TMZ sensitization, impaired GSC self-renewal	Menezes et al. (2019), Rampazzo et al. (2022), Kim et al. (2004)
Panobinostat (LBH589)	Class I, II and IV HDACs	GSC lines, NCH644 NCH421k and U87-MG	Radiosensitization	Tung et al. (2018), Nguyen et al. (2020)
Valproic acid	HDAC1/2	GSC lines	Radiosensitization	Riva et al. (2016)
		Phase II clinical trial - combination therapy with TMZ, and radiotherapy	Active in newly diagnosed GBM in adults	Krauze et al. (2015)
JOC1	HDAC6	GBM lines - U87-MG, U373-MG, U251-MG, A172, T98-G, GNS166 and GNS179	Reduction of GSC proliferation and self-renewal	Auzmendi-Iriarte et al. (2020)
MS-275	Class I HDAC	U87MG and U251, combination therapy with TAK-733 or trametinib	Inhibition of sphere formation, decrease in expression of GSC markers	Essien et al. (2022)
Domatinostat (4SC-202)	Class I HDAC	GS-Y01, GS-Y03 and TGS01	Inhibition of GSC growth	Nakagawa-Saito et al. (2022)
Givinostat	HDAC (Unknown)	GSC lines	Enhancing TMZ Sensitivity, inhibition of MGMT expression in GSCs	Nakagawa-Saito et al. (2023)
C646	P300/CBP	GSC and Xenograft models	Reduced vascular-like phenotype conversion of GSCs and enhanced radiation sensitivity	Muthukrishnan et al. (2022)
CPI-1612	P300/CBP	Primary GBM lines and Xenograft models	Enhancing TMZ Sensitivity	Mladek et al. (2022)

## Histone methyltransferases and histone demethylases

Histone methyltransferases (HMTs) such as lysine methyltransferase (KMTs) and arginine methyltransferase (PRMTs) catalyze the addition of methyl groups on lysine and arginine residues on histones, particularly on H3 and H4 (Greer and Shi, 2012). Histone demethylases (HDMs), which remove methyl groups on histones, are categorized into two groups: the amino oxidase homolog lysine demethylases (KDMs) and the JmjC domain-containing histone demethylases (D’Oto et al., 2016). G9a, a histone methyltransferase is highly expressed in GBM cells and sensitizes tumors to chemo-radiation treatment (Ciechomska et al., 2018). Of the PRMTs, PRMT2 expression was eleveated in GBM and shown to be essential for GSC renewal and GBM tumorigenesis through methylation of H3R8me2a and activation of oncogenic transcriptional programs (Dong et al., 2018). PRMT6 promotes tumorigenicity and radiation response in GSC through methylation of regulator of chromatin condensation 1 (RCC1) signaling (Huang et al., 2021). The SET domain and mariner transposase fusion gene (SEMTAR), an HMT responsible for H3K36 methylation, contributes to radiation resistance by recruiting Ku80 DNA damage repair protein, facilitating the nonhomologous end joining (NHEJ) repair pathway. This recruitment enhances DNA repair, enabling survival of residual GBM cells after radiation (Kaur et al., 2020).

Histone demthylases are overexpressed in GBM and influence tumorigenicity and chemotherapy resistance. KDM5A and KDM6B are implicated in mediating TMZ resistance (Romani et al., 2019). Inhibition of KDM4C and KDM7A activity in GSCs induces DNA damage and disrupts the stem cell-like chromatin state, leading to GSC differentiation into a more differentiated, non-proliferative state. This differentiation is linked to a loss of self-renewal capacity, enhancing GSC response to DNA-damaging therapies (Mallm et al., 2020). Furthemore, Lysine-specific demethylase 1 (LSD1/KDM1A) promotes self-renewal of GSC and TMZ resistance (Alejo et al., 2023). EZH2 (Enhancer of Zeste homolog 2), a catalytic subunit of the PRC2 complex and a H3K27 methyltransferase, has been extensively investigated in GBM and plays a key role in promoting GSC stemness and TMZ resistance (Sharma et al., 2017; Yu et al., 2023). Inhibitors targeting EZH2 including UNC1999, GSK343, GSK126, tazemetostat and CPI-1205 have shown potent effects in blocking PMT in GBM (Yu et al., 2017; Grinshtein et al., 2016). A summary of HMTs and HDMs inhibitors evaluated in GBM are presented in Table 2.

**TABLE 2:** A summary of HMTs and HDMs inhibitors tested in GBM.

Inhibitors	Targets	Cell lines/Models	Outcomes	References
Tranylcypromine, NCL-1 and NCD-38, GSK-14, SD70, JIB-04, GSKJ4, DMOG, NCD38, GSK-LSD1	KDMs: KDM1A, KDM class/KDM2B, KDM4C, KDM class/KDM5A, KDM6B, KDMs 2-7	LN-18, U87MG, U251, GSC lines	TMZ sensitivity	Alejo et al. (2023), Sareddy et al. (2017), Singh et al. (2011), Staberg et al. (2018), Banelli et al. (2017), Romani et al. (2019), Stitzlein et al. (2023)
MC4040 and MC4041	EZH2	GBM primary cells	Reversed PMT	Stazi et al. (2019)
DZNep, GSK126 and AC1Q3QWB	EZH2	U87MG, U251, LN-18, LN229, D54, GSC lines, murine GBM models	Decrease in GSC self-renewal, TMZ resistance	Li et al. (2019c), Ratnam et al. (2021), Tan et al. (2007)
BIX01294	EHMT2 (G9a)	U87MG, WG4, LN18, L0125, L0627	Decrease in GSC self-renewal, TMZ resistance	Ciechomska et al. (2016), Alexanian and Brannon (2022)

## BET proteins

Bromodomain and Extraterminal Domain (BET) proteins recognize lysine-acetylated histones and function as epigenetic readers that regulate transcription (Belkina and Denis, 2012). These proteins including BRD2, BRD3, BRD4 and BRDT have emerged as promising anticancer targets in a broad spectrum of human malignancies including GBM. Among BET proteins, BRD4 was shown to be critical regulator of GSC stemness and tumorigenicity via regulation of NOTCH1 promoter (Tao et al., 2020). Inhibition of BET proteins sensitizes GBM cells to TMZ by reducing the MGMT expression (Tancredi et al., 2022). Several BET inhibitors, such as JQ1 and ZBC260 have been shown to reduce GSC stemness and PMT and improving TMZ sensitivity (Colardo et al., 2023; Duan et al., 2023). Furthermore, OTX015 (MK-8628), a novel inhibitor targeting BRD2/3/4 was reported to exhibit significant anti-tumor effects in combination with TMZ in orthotopic xenograft models (Berenguer‐Daizé et al., 2016). However, it is worth noting that while BET proteins are attractive therapeutic targets for combinatiorial therapies in GBM, the lack of selective inhibitors targeting individual members of the BET family has slowed their development in clinical trials.

## Other histone PTMs

The understudied modifications such as ubiquitination, sumoylation, phosphorylation, palmitoylation, succinylation and lactylation are being increasingly recognized as critical regulators of GBM resistance and progression. Abnormal histone ubiquitination patterns have been found to be associated with poor prognosis and enhanced survival in GBM (Jeusset and McManus, 2019). For example, BMI1-mediated ubiquitination of histone H2A at lysine 119 (H2AK119ub1) leads to transcriptional repression of tumor suppressor genes and contributes to the self-renewal and proliferation of GSCs (Kong et al., 2018). Several deubiquitinating enzymes (DUBs) such as USP1, USP3, USP4 and USP22, are also implicated in GBM. USP1 is overexpressed in GSCs and has been shown to enhance the radiosensitivity of GBM cells (Kong et al., 2018). Phosphorylation of histone H3 at sites such as H3T6 and H3S10 are associated with increased DNA damage repair capacity and resistance to TMZ and radiation therapy (Pacaud et al., 2015). Inhibition of histone phosphorylation using enzastaurin has been shown to increase GBM sensitivity to chemo-radiation therapy (Pacaud et al., 2015). Histone lactylation, facilitated by lactyltransferases, involves the transfer of lactate from lactyl-CoA to histones (Lu et al., 2024). Specifically, histone H3K9 lactylation (H3K9la) drives TMZ resistance in GBM by activating LUC7L2, which reduces MLH1 expression and impairs mismatch repair. Targeting lactylation with stiripentol, an LDHA/B inhibitor, was shown to restore TMZ sensitivity in resistant GBM cells (Yue et al., 2024). Together, these studies underscore the importance of investigating less common histone PTMs in mediating GBM plasticity and resistance, which can open new avenues for therapeutic targeting.

## Non-coding RNAs

Approximately 70% of the genome can generate non-coding RNAs (ncRNAs), which include long-ncRNAs (lncRNA), miRNAs and circRNAs and regulate the translation of messenger RNAs to functional proteins. All ncRNAs, especially miRNAs and lncRNAs are dysregulated in GBM and play key roles in driving plasticity and resistance (Shahzad et al., 2021). While there are still gaps in understanding how miRNAs interact with lncRNAs, these ncRNAs may serve as predictive or prognostic biomarkers and novel therapeutic targets for improving GBM outcomes (Mousavi et al., 2022). Here, we will mainly focus on miRNAs and lncRNAs implicated in GBM resistance (Figure 2).

**FIGURE 2:**
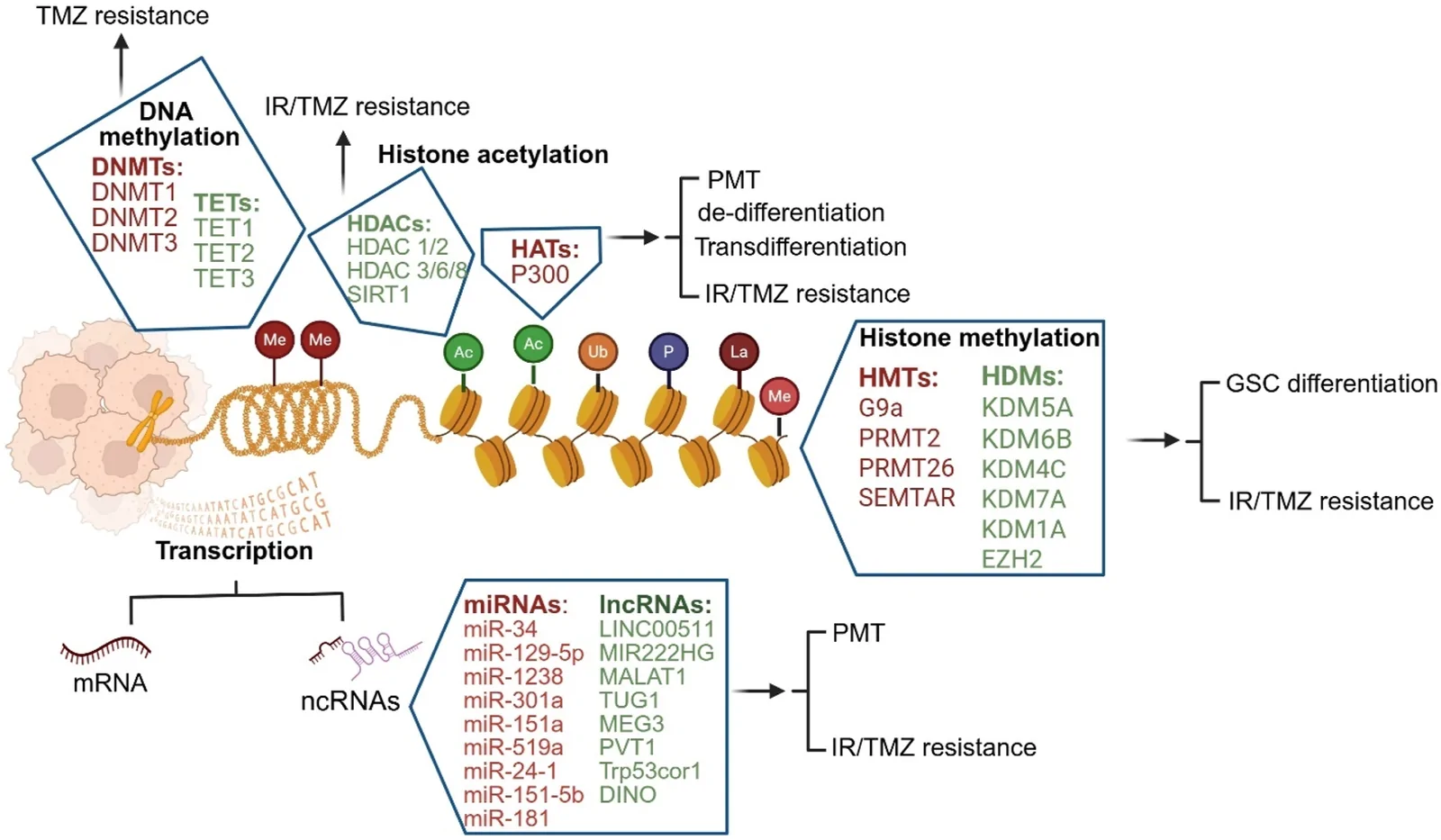
Epigenetic mechanisms mediating GBM plasticity and resistance. DNA methylation, histone modifications such as methylation, acetylation, ubiquitination, phosphorylation, and lactylation, as well as non-coding RNAs are the most well-characterized epigenetic mechanisms in GBM (DNMT- DNA methyltransferases, TET- Ten-eleven translocation, HDAC- Histone deacetylases, HAT- Histone acetyltransferases, HDM- Histone demethylases, HMT- Histone methyltransferases, ncRNA-non-coding RNA, miRNA-micro-RNA, siRNA-small interference RNA, piRNA- Piwi-interacting RNA).

### miRNAs

miRNAs are small ncRNAs that typically consist of 20–22 nucleotides. They act as both tumor suppressors and onco-miRNAs and are highly dysregulated in GBM. miRNAs are predominantly involved in mediating chemo-radiation resistance and PMT. MiR-34 enhances GSC self-renewal, and therapeutic resistance by targeting EGFR signaling (Yin et al., 2013). MiR-129-5p promotes TMZ resistance by targeting DNMT3a (Gu et al., 2018). Moreover, miRNAs such as miR-1238 promotes resistance to TMZ via targeting CAV1/EGFR pathway, while miR-301a promotes resistance through regulating BTG1 (Yin et al., 2019; Xiao et al., 2021), while others, like miR-151a and miR-519a, enhance chemosensitivity by targeting STAT3/Bcl2 signaling pathway (Zeng et al., 2018; Li et al., 2018). Several other miRNAs are implicated in modulating the radiation-response of GBM. Overexpression of miR-24-1 and miR-151-5b, following radiation, reduces the expression of the tumor suppressor PDCD4, promoting resistance (Chao et al., 2013; Mukherjee et al., 2022; Sufianov et al., 2022). Additionally, miR-181a sensitizes GBM cells to radiation by targeting Bcl-2, while miR-301a, secreted in exosomes by hypoxic cells, activates Wnt/β-catenin signaling to enhance radiation resistance (Chen et al., 2010; Yue et al., 2019).

### Long-non coding RNAs

Long non-coding RNAs (lncRNAs) are a diverse group of RNA molecules typically longer than 200 nucleotides. They constitute over 80% of total non-coding RNAs and play crucial roles in regulation of gene expression, chromatin remodeling and cell signaling (Zhang et al., 2019). In the context of GBM, lncRNAs are key epigenetic regulators that influence tumor biology, including plasticity and resistance (Doghish et al., 2025). Several lncRNAs have been shown to directly affect the malignant characteristics of GSCs (Stackhouse et al., 2020; de Oliveira et al., 2019; Wang et al., 2023). For example, LINC00511 and MIR222HG induce PMT, which is associated with increased tumor aggressiveness, therapeutic resistance and worse prognosis for patients (Fan et al., 2023; Azam et al., 2020). MALAT1 regulates the PI3K/Akt pathway and promotes TMZ resistance (Cai et al., 2018). Other lncRNAs, such as TUG1 and MEG3, contribute to drug resistance by regulating drug efflux pumps and metabolic pathways that help GBM cells evade treatment (Luo et al., 2021). PVT1, Trp53cor1, TUG1 and DINO activate the p53-dependent DNA repair pathways to enhance GBM survival after radiation (Aryankalayil et al., 2018). Targeting these miRNAs and lncRNAs represent a promising approach to combat GBM resistance.

## Challenges and opportunities in targeting epigenetic modifiers in GBM

Although fractionated radiation and TMZ chemotherapy continue to be the standard of care for GBM, the benefits are short-lived as tumors invariably develop resistance. Since epigenetic modifiers play a crucial role in the acquisition of phenotypic plasticity and resistance, a better understanding of their functions and mechanisms is vital to develop effective therapeutic modalities for GBM. Future investigations should focus on determining the underlying molecular mechanisms by which DNA methylation, histone modifications and ncRNAs influence GSC plasticity and resistance; as well as how their dynamics are altered by environmental stressors.

Targeting epigenetic modifiers with small molecule inhibitors remains a key challenge for GBM. There are several barriers that need to be addressed including: a) the inability of the inhibitors to cross the blood-brain-barrier and penetrate the tumors, b) lack of selectivity to target tumor cells, while sparing normal cells, c) lack of substrate specificity and off-target effects and d) unanticipated cytotoxicity. It is also imperative that more research is needed to develop rational pre-clinical models and drug delivery strategies to tackle these challenges. PROTACs (Proteolysis Targeting Chimeras) technology has emerged as a powerful strategy for drug delivery selectively targeting histone modifier enzymes. By harnessing the ubiquitin-proteasome system, PROTACs induce the selective degradation of these enzymes, allowing for the modulation of epigenetic regulation with high specificity (Rutherford and McManus, 2024; Alhasan et al., 2024). For instance, SPP-ARV-825 nanosystem combines the BRD4-degrading PROTAC ARV-825 with a micelle designed to cross the blood-brain barrier. This system effectively reduces tumor cell proliferation, induces apoptosis and inhibits tumor-associated macrophage polarization in GBM (Yang et al., 2022). The PROTAC-like inhibitor J22352 targets HDAC6 for degradation and significantly inhibited GBM progression by enhancing autophagic cell death and activating anti-tumor immunity (Liu et al., 2019). Moreover, nucleotide- and RNAi-based approaches such as antisense oligonucleotides (ASOs), locked nucleic acids (LNAs), peptide nucleic acids (PNAs), morpholino oligonucleotides (MO), miRNA mimics and antagomirs, short interfering RNAs (siRNAs) and short hairpin RNAs (shRNAs) are also an alternative and viable therapeutic strategy. Nanoparticles (NPs) have emerged as a promising approach to deliver chemotherapeutics and RNAi molecules as they increase their lifespan in circulation and improve their cellular uptake and endosomal escape. Several types of NPs have been tested for systemic delivery of RNAi-based molecules and chemotherapeutics including liposomes, polymeric NPs, micelles, dendrimers, artificial DNA nanostructures, silica NPs, nanotubes, metal NPs and quantum dots enabling drug delivery to the brain (Lopez-Bertoni et al., 2018). These novel and emerging approaches have the potential to overcome the limitations associated with delivery, target specificity and safety and need to be further validated in pre-clinical models for their anti-tumor efficacy prior to clinical translation.

## Conclusions and future directions

Our review has highlighted the functional significance of epigenetic modifications in driving GBM plasticity and resistance. By elucidating the epigenetic landscape of GBM, we can potentially identify novel biomarkers, improve patient stratification and design rational and personalized treatment strategies for better clinical outcomes. In order for pre-clinical findings to translate into successful clinical trials, chemotherapeutics will need to be tested on humanized animal models and patient-derived organoid models that more closely mimic patient tumors. Moreover, leveraging multi-omics technologies including single-cell and spatial transcriptomics, epigenomics, proteomics and metabolomics combined with histology and functional imaging can potentially reveal how GBM tumors evolve across space and time, their adaptation to different environments and therapeutic insults, unravel novel cellular and molecular interactions and identify novel mechanisms driving plasticity, resistance and recurrence.

## Abbreviations

GBM, Glioblastoma; TMZ, Temozolomide; GSCs, GBM stem-like cells; EC, Endothelial-like cells; PC, Pericyte-like cells; PN, Proneural; MES, Mesenchymal; PMT, Proneural to mesenchymal transition; EMT, Epithelial-mesenchymal transition; TME, Tumor microenvironment; ncRNAs, Non-coding RNAs; NPC, Neural progenitor-like cells; OPC, Oligodendrocyte progenitor-like cells; AC, Astrocyte-like cells; PDOX, Patient-derived orthotopic xenograft; ATAC, Assay for Transposase-Accessible Chromatin; CAFs, Cancer-associated fibroblasts; 5mC, 5-methylcytosine; DNMTs, DNA methyltransferases; CFP1, CpG binding protein 1; TET, Ten-eleven translocation; 5-mdC, 5-methyl deoxycytosine; 5-hmdC, 5-hydroxymethyl-2′deoxycytidine; 5-fdC, 5-formly-2′deoxycytidine; 5-cadC, 5-carboxyl-2′deoxycytidine; PRC2, Polycomb repressive complex protein 2; DNMTis, DNA methyltransferase inhibitors; PTMs, Post-translational modifications; HDACs, Histone deacetylases; HATs, Histone acetyltransferases; KATs, Lysine/histone acetyltransferases; GNAT, Gcn5-related N-acetyltransferase; TAFII250, TATA-binding protein-associated factor 250; TFIIIC, Transcription factor IIIC; HIF2A, Hypoxia-inducible factor 2A; HDACis, Histone deacetylase inhibitors; HMTs, Histone methyltransferases; KMTs, Lysine methyltransferases; PRMTs, Arginine methyltransferases; HDMs, Histone demethylases; KDMs, Lysine demethylases; EZH2, Enhancer of zeste homolog 2; BET, Bromodomain and extraterminal domain; MGMT, O-6-methylguanine-DNA methyltransferase; NHEJ, non-homologous end joining; lncRNA, Long non-coding RNA; miRNAs, MicroRNAs; TCGA, The Cancer Genome Atlas; MMPs, Matrix metalloproteinases; ASOs, Antisense oligonucleotides; LNAs, Locked nucleic acids; PNAs, Peptide nucleic acids; MO, Morpholino oligonucleotide; siRNAs, Short interfering RNAs; shRNAs, Short hairpin RNAs; NPs, Nanoparticles.
